# Effectiveness of two vocational interventions on sickness absence and costs for people with musculoskeletal disorders: 12 months results from the MI-NAV multi-arm randomized trial

**DOI:** 10.5271/sjweh.4248

**Published:** 2025-11-01

**Authors:** Britt Elin Øiestad, Esther Maas, Fiona Aanesen, Alexander Tingulstad, Tarjei Rysstad, Maurits van Tulder, Anne Therese Tveter, Milada Hagen, Rigmor C Berg, Nadine E Foster, Gwenllian Wynne-Jones, Gail Sowden, Gunnhild Bagøien, Roger Hagen, Kjersti Storheim, Margreth Grotle

**Affiliations:** 1 Department of Rehabilitation science and health technology, Oslo Metropolitan University, Oslo, Norway.; 2 Department of Health Sciences, Faculty of Science, Vrije Universiteit Amsterdam, and the Amsterdam Movement Sciences Research Institute, BT Amsterdam, The Netherlands.; 3 National Institute of Occupational Health, Oslo, Norway.; 4 Faculty Behavioral and Movement Sciences, Vrije Universiteit Amsterdam, BT Amsterdam, The Netherlands.; 5 Center for treatment of rheumatic and musculoskeletal diseases (REMEDY), Diakonhjemmet Hospital, Oslo.; 6 Department of Nursing and Health Promotion, Oslo Metropolitan University, Oslo, Norway.; 7 Norwegian Institute of Public Health, Oslo, Norway, and The Arctic University of Norway, Tromsø, Norway.; 8 STARS Education and Research Alliance, Surgical Treatment and Rehabilitation Service (STARS), The University of Queensland and Metro North Health, Brisbane, Australia.; 9 School of Medicine, Keele University, Keele, UK.; 10 Connect Health, Newcastle upon Tyne, Tyne and Wear, UK.; 11 Nidelv Community Mental Health Center, Tiller, Clinic of Mental Health, St Olavs University Hospital, Trondheim, Norway.; 12 Department of Psychology, University of Oslo, Department of Psychology, Norwegian University of Science and Technology, Research Institute, Modum Bad, Norway.; 13 Division of Clinical Neuroscience, Department of Research and Innovation, Oslo University Hospital, Oslo, Norway.; 14 Centre for Intelligent Musculoskeletal Health, Oslo Metropolitan University, Oslo, Norway.

**Keywords:** case management, motivational interviewing, return to work, stratified vocational advice intervention, vocational rehabilitation

## Abstract

**Objectives:**

This study aimed to assess 12-month outcomes on return to work (RTW) and cost-effectiveness in adults on sick leave due to musculoskeletal disorders who were randomized to either usual case management (UC), UC+motivational interviewing (MI) or UC+stratified vocational advice intervention (SVAI).

**Methods:**

The study was conducted in the Norwegian Labor and Welfare Administration (NAV). Workers on sick leave due to musculoskeletal disorders for ≥50% of their contracted work hours for ≥7 consecutive weeks were included. Trained case workers delivered MI in two face-to-face sessions, and physiotherapists provided SVAI and identified RTW obstacles. The main outcomes were sick leave days over 12 months and cost-effectiveness, cost-utility and cost-benefit.

**Results:**

The trial included 509 workers with a mean age of 48 years. There were statistically significant differences between UC+MI versus UC [-15.6 days, 95% confidence interval (CI) -31.0– -0.2], and UC+SVAI versus UC (-17.6 days, 95% CI -33.0– -2.2). Compared to UC, odds ratios (OR) for receiving wage replacement benefits each month were lower for UC+MI (OR=0.73, 95% CI 0.64–0.84), and UC+SVAI (OR 0.74, 95% CI 0.64–0.84). The probabilities of cost-effectiveness were high for adding either MI or SVAI to UC (ceiling ratio 0.90), and the net benefit for MI was €5225 (95% CI -592–10 985) and for SVAI €7214 ((95% CI 1548–12 851) per person.

**Conclusions:**

Adding MI or SVAI to UC significantly improved RTW outcomes and was cost-effective among people on sickness absence due to musculoskeletal disorders.

Sickness absence poses a significant challenge for individuals, employers and society at large. It is associated with reduced productivity, increased healthcare utilization and substantial social security expenditure (1). While time off from work may support recovery in the short term, accumulating evidence suggests that returning to work can promote health (2) and well-being (3).

In Norway, 5–7% of the workforce is consistently on sick leave (4). The long-term sickness absence of >8 weeks is around 3%, mainly caused by musculoskeletal (eg, neck and back pain) and mental disorders (eg, anxiety and depression) (5). Individuals on sick leave receive full wage compensation for up to 12 months, and the follow-up process involves several stakeholders. Sick leave is generally certified by general practitioners (GP), while the employers are responsible for early follow-up. Employers cover wage replacement for the first 16 days of absence, after which the Norwegian Welfare and Labor Administration (NAV) assumes responsibility for wage compensation up to 12 months. NAV also coordinates a dialogue meeting within 26 weeks, involving the employee, employer, and GP.

Beyond this usual case management (UC), many NAV offices offer various approaches to help people return to work (RTW). One approach is motivational interviewing (MI), a method addressing motivation for behavioral change (6). MI is a person-centered and collaborative counseling style used in a variety of settings, such as increasing treatment adherence for people with musculoskeletal pain conditions (7, 8). MI has been tested in a broad range of diseases and to facilitate lifestyle changes (9, 10), but there is a lack of scientific evidence concerning the effectiveness of MI in facilitating RTW (11, 12). Considering the positive results in other diseases – and that many NAV offices have implemented MI in their practice – the intervention might work for people on sick leave due to musculoskeletal disorders. Another approach to facilitate RTW, the stratified vocational advice intervention (SVAI), has shown promising results in reducing sickness absence (13–15). Healthcare providers (eg, a physiotherapist) deliver SVAI in a dialogue meeting with the person on sick leave, with the aim to reveal and overcome obstacles for RTW (15). Both MI and SVAI are low-intensity interventions that might have the potential to reduce long-term sick leave among people with musculoskeletal disorders.

Recently, we conducted a three-arm randomized controlled trial (RCT) to evaluate work, health, and cost-effectiveness (CE) outcomes among people on sick leave due to musculoskeletal disorders (the MI-NAV trial) (14, 16). At the 6-month follow-up, analyses showed promising results on sickness absence days when adding MI to UC [-7 days, 95% confidence interval (CI) -15–2] and when adding SVAI to UC (-7 days, 95% CI -15–1) compared to UC alone (14). Furthermore, our 6-month CE, -utility and -benefit analyses showed that MI and SVAI potentially could reduce the societal costs related to sickness absence (17). Here we report the 12-month differences between the groups on sickness absence days, time until RTW, receiving wage replacement benefits each month, self-reported musculoskeletal health, and CE in the MI-NAV trial.

## Method

### Design

This study was a three-arm parallel RCT with 12 months of follow-up reporting the secondary outcomes. The trial protocol (16), process evaluations (15, 18), and the 6-month results have been published (14, 17, 19, 20). The trial is reported according to the Consolidated Standards of Reporting Trials extension statement for reporting multi-arm trials (CONSORT statement) (21) (supplementary material, www.sjweh.fi/article/4248).

### Participants

The NAV delivered weekly lists of all people on sick leave due to a musculoskeletal disorder according to the International Classification of Primary Care (ICPC-2), in one region in the southeastern part of Norway. Eligible participants were workers aged 18–67 years, employed full- or part-time, on sickness absence for ≥50% of their contracted work hours ≥7 consecutive weeks. The workers had to have a ≥20% position. People with serious disorders in need of specialized treatment, pregnant women, unemployed, free-lancers, non-Norwegian, non-English speaking, and self-employed workers were not eligible. Patient user representatives with musculoskeletal disorders were involved in the planning of the trial. They provided guidance related to the relevance of the trial, the aims and how to deliver information to participants.

### Recruitment, stratification, and randomization

Three PhD candidates with a contract and office at the NAV recruited participants by phone from April 2019 to October 2020. Eligible participants were informed about the trial and assured that participation would not affect their wage replacement benefits or the follow-up from NAV (UC). An electronic link to an informed consent and baseline questionnaire was sent to those who were interested. Based on the Örebro Musculoskeletal Pain Screening Questionnaire (ÖMPSQ-SF) (22) and the Keele STarT Musculoskeletal (MSK) Tool (23, 24), we stratified the participants in the SVAI group into a high- or medium/low-risk group for long-term sickness absence (14). Participants with ≥9 on the Keele STarT MSK Tool and ≥60 on the ÖMPSQ-SF were stratified to a high-risk of long-term sick leave group. All others were stratified to a medium/low-risk of long-term sick leave group. A statistician prepared a computer-generated allocation sequence. Random allocation was conducted within each stratum of risk group (1:1:1), concealed for the recruitment staff and the other researchers.

### Interventions

UC, MI and SVAI are described in detail in Appendix I to the study by Aanesen et al (14).

### Usual case management

All participants got UC including full wage replacement benefits for up to 12 months after receiving a sick note, and all participants who had not returned to work by week 26 could receive a NAV dialogue meeting arranged if needed.

### Motivational interviewing

A NAV case worker delivered the MI intervention face-to-face as soon as possible after the recruitment date, and a second session was conducted two weeks later. Prior to the trial onset, the case worker received six days of MI training (3 + 2 + 1 days) and an intervention manual. In addition, the case worker received supervision every second month throughout the trial. MI was based on the assumption that people have the resources within themselves to change according to Miller & Rollnick (6). A key factor in MI is that change is activated through the person’s own talk about their reasons and needs for changes towards a goal. In our study, these changes concerned their sick leave and the RTW process where also the persons’ work tasks and possible NAV RTW assistance were addressed. The case worker followed guidelines including MI principles to build a collaborative relationship with the participants, including open-ended questions, providing reflections and summaries to evoke and enhance change talk. Readiness for RTW was a key factor during the conversations. Fidelity of the MI intervention has previously been described (18).

### Stratified vocational advice intervention

Four trained physiotherapists delivered the SVAI intervention as soon as possible after the recruitment. Participants stratified to the high-risk group received 3–4 phone calls. The low/medium-risk group received 1–2 phone calls. The physiotherapists followed a semi-structured conversation guide with open-ended questions to clarify the participants’ work and health situation and identify obstacles to RTW. The physiotherapists provided evidence-based advice on treatment of musculoskeletal disorders and supported problem-solving to overcome modifiable obstacles. SVAI was tailored to decide goals for RTW, develop and implement action plans, and facilitate communication, collaboration and coordination with other services if needed.

### The outcomes

This trial reports the following outcomes from 12 months of follow-up: (i) sickness absence days from baseline to 12 months, (ii) time (months) until sustained RTW, defined as the first 4-week period of 50–100% return to contracted work hours without relapse, (iii) receiving wage replacement benefits each month, (iv) musculoskeletal health, (v) quality-adjusted life years (QALY), and (vi) costs-effectiveness, cost-utility, and cost-benefit.

Data on sickness absence was retrieved from national registries and included the actual days away from work adjusted for contracted work hours. Musculoskeletal health was assessed at the 3-, 6-, 9-, and 12-month follow-ups using the Musculoskeletal Health Questionnaire (MSK- HQ) (25, 26). QALY were measured at the 12-month follow-up by the EuroQol-5 Dimentions-5 Levels (EQ-5D-5L) (27). Baseline characteristics and data on potential confounding factors were collected through questionnaires distributed electronically at baseline (16, 24), including age, gender, body mass index (based on self-reported height and weight), education level, marital status, first language, smoking (yes/no), physical activity [single question from the MSK-HQ), workability (single question from the Work Ability Index, 0-10 scale (28)], follow-up from employer (yes/no), sickness absence percentage, sickness absence days the year prior to inclusion, and days of sickness absence at baseline.

Information on healthcare costs was retrieved from national registers, namely, the Norwegian Health Economics Administration and the Norwegian Patient Registry. Indirect costs consisted of work absenteeism and productivity losses due to paid and unpaid work. We obtained absenteeism data from national registries and valued it using estimates from official statistics on average income stratified by gender. Productivity losses due to unpaid work were measured using the Institute for Medical Technology Assessment Productivity Cost Questionnaire (iPCQ) (29). All costs were converted to 2021 euros.

### Sample size

Sample size was calculated for two-arm comparisons with an expected 10-day mean difference (standard deviation of 28 days) between the intervention groups and UC at 6-months (14), derived from results of the UK SWAP trial (13) and a work-related trial in Sweden (30). With a statistical power of 80% and a two-sided 5% significance level, each arm required 125 participants. We expected 5% loss to follow-up and included 450 participants.

### Statistical analyses of data

The analyses were performed according to the intention-to-treat principle (31), and single models were used for the multiple group analyses (ClinicalTrials.gov identifier: NCT03871712).

The variable *sickness absence days* was reported with the median and quartile 1 and 3 (Q1-Q3). Group differences were analyzed with the Mann–Whitney U test and with unadjusted and adjusted robust linear regression models. We preplanned to include the following potential confounding factors if the groups were unbalanced at baseline: age, sex, education level, sickness absence previous year, workability, musculoskeletal health, physical activity, and employer follow-up. We conducted sensitivity analyses with multiple imputation by chained equations for this research question, with 10 datasets, to account for missing data (32). Imputed baseline variables were workability (missing N=3), physical activity level (missing N=1), musculoskeletal health (missing N=21), follow-up from employer (missing N=7).

Probabilities for *time until sustained RTW* are presented with 95% confidence intervals (CI). Unadjusted differences in *time until sustained RTW* were analyzed with Kaplan Meier method and the groups were compared using the log rank test. Curves, and P-values from the log-rank tests, are presented. Adjusted analyses were conducted using Cox proportional hazard model with all three groups included in the model. The results are expressed as hazard ratios (HR) with 95% CI. Unadjusted and adjusted group differences for *receiving wage replacement benefits each month* were analyzed with generalized linear model (GLM) with logit link. The results are presented graphically and reported as the odds ratio (OR) for receiving benefits in each intervention arm compared to UC.

Unadjusted and adjusted differences between the intervention groups and the UC group on *musculoskeletal health* were analyzed using linear mixed-effect models for repeated measures. The fixed effects in the model were intervention group, time of measurement, and baseline score. The effect of the interventions at each follow-up was estimated with the interaction term: group×time. Random intercepts were incorporated into the model to account for the dependence of repeated measures. Unstructured covariance matrix was used in the model. The results are expressed as the estimated differences in points from MSK-HQ between the intervention groups versus UC.

### Cost-effectiveness, cost-utility, and cost-benefit

This study adopted a societal perspective, including direct and indirect costs. Direct costs included costs of the intervention, primary healthcare use, and secondary healthcare use. A micro-costing approach, including training and mentoring costs, was used to calculate intervention costs. Missing data were imputed using multivariate imputation by chained equations (MICE) with predictive mean matching (32). Ten complete datasets were imputed, and analyses were performed per imputed dataset separately. The results were then pooled using Rubin’s rules (32). The outcome measure in the CE analyses was sickness absence days, and hence productivity cost was not included to avoid double counting. The outcome measure for the cost-utility analysis was health gains, expressed as QALY, derived from the EQ-5D-5L utility scores at the 12-month follow-up.

Unadjusted and adjusted linear regression models were conducted to analyze disaggregate cost differences. Differences in total costs and effects between treatment groups were obtained from a system of seemingly unrelated regressions (SUR) that accounted for the potential correlation between costs and effects (33). The incremental CE ratio (ICER) was calculated for each of the intervention groups compared to the UC group, defined by the incremental costs relative to QALYs gained. To deal with the highly skewed cost data, uncertainty around costs and effects was analyzed using the bootstrap method with 10 000 replicated datasets. To illustrate the statistical uncertainty surrounding the ICER, the bootstrapped cost and effect pairs were plotted on a CE plane with the ICER on the y-axis and the incremental effects on the x-axis.

CE acceptability curves were constructed that indicate the probability of UC+MI and UC+SVAI being cost-effective compared to UC at different levels of willingness-to-pay. The latter is the maximum amount of money decision makers are willing to pay for the additional unit of effect (QALY and sickness absence) (35). A cost-benefit analysis was performed. Three ROI metrics were calculated (i); net benefit (NB), (ii) benefit-to-cost ratio (BCR), and (iii) return on investment (ROI).

- *NB = benefits − costs*
- *BCR = benefits/costs*
- *ROI = (benefits − costs)/costs [*×*100]*

Costs were defined as intervention costs. Benefits were defined as the difference in monetized outcome measures (ie, absenteeism, and presenteeism costs) between the intervention and control groups during follow-up, with positive benefits indicating reduced spending. To test the robustness of the results, we conducted complete-case analysis, and analyses using the unimputed dataset.

## Results

### Enrolment

Of 514 participants randomized five participants withdrew their consent, leaving 509 participants. A flow diagram of participants is shown in supplementary figure S1. The first MI and SVAI sessions were given on average 21 (13) and 6 (5) days after the recruitment date.

Characteristics of the participants are listed in table 1. At baseline, over one-third of the participants had full sickness absence. Baseline characteristics were overall balanced across the groups, but there were slightly fewer women, slightly fewer with high education, and more participants with lower activity level in the UC group. The year prior to baseline, the UC group had median (min–max) 38 (1–152) sickness absence days with the corresponding numbers for UC+MI of 35 (6–191) and for MI+SVAI 36 (4–200). Median sickness absence days at baseline were for UC 51 (0–234), for UC+MI 51 (0–344) and for UC+SVAI 51 (0–372).

**Table 1:** Baseline characteristics of the study participants across the three groups. [N=number; SD=standard deviation; UC=usual case management; MI=motivational interviewing; SVAI=stratified vocational advice intervention].

Variable		UC (N=171)					UC+MI (N=169)					UC+SVAI (N=169)			
		N	%	Mean	SD		N	%	Mean	SD		N	%	Mean	SD
Age				47.3	10				48.3	10.5				47.9	10
Body mass index (kg/m^2^)				28.2	5.1				28.3	5.8				28.1	5.4
Workability (0–10)				2.8	2.5				3.2	2.7				3.1	2.6
Musculoskeletal health (0-56)															
	Baseline (N=488)			27	9				27	8				27	8
	3 months (N=373)			32	10				33	10				34	10
	6 months (N=333)			34	10				34	11				36	11
	9 months (N=295)			34	11				37	11				37	12
	12 months (N=295)			34	11				36	11				37	11
Women		93	54				99	59				99	59		
Higher education (college/university)		60	35				61	36				66	39		
Married or living with partner		118	69				118	70				119	70		
Norwegian as first language		150	88				154	85				144	85		
Smoking		38	22				35	21				36	21		
Physical activity level previous week															
	Low (0–2 days)	109	64				96	57				103	61		
	High (3–7 days)	62	36				72	43				66	39		
Full sick leave of contracted work hours		103	60				109	64				103	60		
Part time sick leave of contracted work hours *		65	38				56	33				64	38		
Area of body pain **															
	Lower limb	6	1.2				18	3.6				15	3		
	Upper limb	30	6				30	6				30	6		
	Neck	12	2.4				12	2.4				10	2		
	Back	34	6.8				42	8.4				43	8.6		
	Multisite pain	12	2.4				8	1.6				10	2		
	Joint disorders	20	4				13	6.2				10	2		
	Fractures	14	2.8				16	3.2				11	2.2		
	Others	40	8				26	5.2				38	7.6		
White-collar workers		57	11.2				56	11				61	12		
Blue-collar workers		114	22.4				113	22.2				108	21.2		

* 9 participants evenly distributed across the three groups had already returned to work at the baseline measurement. ** 9 patients had missing data.

### Sickness absence days at 12 months

At the 12-month follow-up, the median (Q1–Q3) sickness absence from baseline for the UC group was 85 (40–151) days, 73 (29–136) days for UC+MI, and 69 (32–120) days for UC+SVAI (table 2). Unadjusted median comparisons showed no statistically significant difference in sickness absence days between UC+MI and UC, but UC+SVAI had statistically significant fewer sickness absence days compared to UC. In the adjusted analyses (N=479) there was a statistically significant difference between UC+MI versus UC (-15.6 days, 95% CI -31.0– -0.2), and between UC+SVAI versus UC (-17.6 days, 95% CI -33.0– -2.2).

**Table 2:** Descriptive, unadjusted results for outcomes over 12 months. [UC=usual case management; MI=motivational interviewing; SVAI=stratified vocational advice intervention; RTW=return to work; CI=confidence interval].

Variable	UC (N=171)	UC+MI (N=169)	UC+SVAI (N=169)
Probability (95% CI) of no sustained RTW for each month	Probability (95% CI)	Probability (95% CI)	Probability (95% CI)
Month 1 (baseline)	0.98 (0.95–0.99)	0.99 (0.95–0.98)	0.99 (0.95–0.98)
Month 2	0.91 (0.85–0.94)	0.82 (0.76–0.88)	0.86 (0.80–0.91)
Month 3	0.82 (0.75–0.87)	0.73 (0.65–0.79)	0.70 (0.62–0.76)
Month 4	0.70 (0.63–0.76)	0.62 (0.54–0.69)	0.62 (0.54–0.69)
Month 5	0.61 (0.54–0.68)	0.53 (0.45–0.60)	0.55 (0.47–0.62)
Month 6	0.57 (0.50–0.64)	0.48 (0.40–0.55)	0.48 (0.40–0.55)
Month 7	0.54 (0.47–0.62)	0.42 (0.35–0.49)	0.40 (0.32–0.47)
Month 8	0.50 (0.42–0.57)	0.38 (0.31–0.45)	0.36 (0.28–0.43)
Month 9	0.38 (0.31–0.45)	0.33 (0.26–0.40)	0.29 (0.22–0.36)
Month 10	0.29 (0.23–0.36)	0.28 (0.21–0.35)	0.25 (0.19–0.32)
Month 11	0.23 (0.17–0.30)	0.21 (0.15–0.28)	0.17 (0.11–0.23)
Month 12	0.19 (0.13–0.25)	0.18 (0.12–0.24)	0.12 (0.08–0.18)

### Time-to-sustained RTW

Months until RTW showed a median (Q1–Q3) of 8 (4–11) for UC, 6 (3–11) for UC+MI and 6 (3–10) for UC+SVAI. The probabilities for time until sustained RTW are showed in table 2 and depicted in supplementary figure S2. The median time until sustained RTW was 8 months (Q1–Q3 4–11) for UC, 6 months (Q1–Q3 3–11) for UC+MI, and 6 months (Q1–Q3 3–10) for UC+SVAI. There was no statistically significant difference in unadjusted time-to-sustained RTW between UC+MI and UC, but a statistically significant difference was found between UC+SVAI and UC (P=0.03). In adjusted analyses, compared to UC, the chance for RTW was higher in the UC+MI (HR 1.17, 95% CI 0.91–1.50) and UC+SVAI (HR 1.27, 95% CI 1.00–1.63).

### Receiving wage replacement benefits

The proportions for receiving wage replacement benefits each month are depicted in supplementary Figure S3. Analyses showed statistically significantly lower odds for MI+UC (unadjusted: OR=0.75, 95% CI 0.66–0.85 and adjusted: OR=0.73, 95% CI 0.64–0.84), and SVAI+UC (unadjusted 0.76, 95% CI 0.67–0.86 and adjusted: 0.74, 95% CI 0.64–0.84) compared to UC.

### Musculoskeletal health

Descriptive data on self-reported musculoskeletal health are shown in table 2. There were no statistically significant differences between the groups over time in the unadjusted and adjusted analyses.

### Cost differences

There were no differences in QALY at 12 months between the three groups with mean (SEM) for UC 0.68 (0.02), for UC+MI 0.71 (0.01) and for UC+SVAI 0.69 (0.01). Mean costs within each trial group are presented in table 3. Total costs were highest in UC with €39 583 [standard error of the mean (SEM) €2122], followed by UC+MI with €34 358 (SEM €2069) and UC+SVAI with €32 368 (SEM €1883). In all three groups, >90% of costs were related to absenteeism and productivity loss. The costs of the interventions were <0.5% of the total costs. Intervention costs were higher in UC+MI and UC+SVAI compared to UC. All other costs were lower for both intervention groups compared to the UC. UC+MI had lower total societal costs in the adjusted analysis with €-5599 (95% CI -11 001–313) compared to UC, this was also the case for UC+SVAI with €-6,505 (95% CI -11 795– -1213).

**Table 3:** Mean cost (€) for 12 months per participant in the various study groups, unadjusted and adjusted mean cost differences between groups. **Total values are depicted in bold font.** [UC=usual case management; MI=motivational interviewing; SVAI=stratified vocational advice intervention; SEM=standard error of the mean; CI=confidence interval].

Cost category		UC (N=171)		UC+MI (N=169)		UC+SVAI (N=169)		Comparison 1: UC+MI vs UC				Comparison 2: UC+SVAI vs UC		
								Unadjusted		Adjusted *		Unadjusted		Adjusted *
		Mean (SEM)		Mean (SEM)		Mean (SEM)		Mean (95% CI)		Mean (95% CI)		Mean (95% CI)		Mean (95% CI)
Intervention costs		0 (0)		53 (3)		78 (3)		53 (47–58)		54 (48–60)		78 (72–84)		78 (72–85)
Healthcare costs														
	Total	1187 (85)		1141 (95)		1049 (98)		-46 (-290–202)		-85 (-340–191)		-138 (-366–160)		-170 (-395–153)
	Primary	1046 (80)		1026 (92)		942 (95)		-20 (-250–218)		-53 (-293–212)		-105 (-317–195)		-128 (-343–198)
	Secondary	141 (14)		114 (11)		108 (11)		-27 (-63–6)		-32 (-73–6)		-34 (-70– -4)		-42 (-82– -6)
Absenteeism costs		35 608 (1963)		31 341 (1963)		29 472 (1782)		-4267 (-9833–979)		-4491 (-9566–989)		-6136(-11 335– -1161)		-5271 (-10 124– -377)
Productivity losses of unpaid work		2787 (470)		1823 (368)		1769 (370)		-965 (-2152–134)		-1076 (-2425– -653)		-1018 (-2187–104)		-1143 (-2355–75)
Total societal costs		**39 583 (2122)**		**34 358 (2069)**		**32 368 (1883)**		**-5225 (-11 185–359)**		**-5599 (-11 001– 313)**		**-7214 (-12 671– -1871)**		**-6505 (-11 795– -1213)**

* Comparisons were adjusted for age, sex, education level, sick leave previous year, workability, musculoskeletal health, risk of work disability (not included in this long-term follow-up), physical activity, and employer follow-up. Results are based on imputed cost values.

### Cost-effectiveness and cost-utility

Comparing UC+MI to UC, we found an ICER of €1 315 801 for QALY, indicating that this sum would, on average, be saved in UC+MI compared to UC per QALY gained. Similarly, for UC+MI, we found an ICER of 97, indicating a saved average of €97 for each day reduction of sickness absence compared to UC. Table 4 and supplementary figure S4 show that most incremental CE pairs were located on the southern (for QALY) and southeast (for sickness absence) quadrant(s) of the CE plane, indicating that UC+MI was on average less costly for improving QALY, and less costly and more effective for reducing sickness absence.

**Table 4:** Cost-effectiveness and cost-utility analysis results (main analysis). [C=costs; E=effect; CI=confidence interval; ICER=incremental cost-effectiveness ratio; CE-plane=cost-effectiveness plane; MI=motivational interviewing; SVAI=stratified vocational advice intervention; UC=usual case management; NE=northeast-quadrant; SE=southeast-quadrant; NW=northwest-quadrant; ZW=southwest-quadrant; QUALY=quality-adjusted life years].

Outcome		Sample size (N)			∆C (95% CI)		∆E (95% CI)		ICER		Distribution CE-plane (%)			
		MI/SVAI	UC		€		Points		EUR/point		NE	SE	SW	NW
Comparison 1 (UC+MI vs. UC)														
	Cost-utility; QALY (0–1)	169	171		-5495 (-11 045–292)		-0.004 (-0.03–0.03)		1 315 801		0.9	59.6	37.6	1.9
	Cost-effectiveness ^1^	169	171		-1110 (-2437–106)		11.4 (-4.1–26.9)		-97		3.5	88.7	7.1	0.7
Comparison 2 (UC+SVAI vs. UC)														
	Cost-utility; QALY (0-1)	169	171		-6604 (-11 897– -1256)		0.003 (-0.03–0.03)		-203 811		0.2	58.2	41.0	0.5
	Cost-effectiveness^1^	169	171		-1293 (-2596–88)		19.2 (4.57–33.87)		-67		2.0	97.5	0.5	0.04

^1^Sickness absence days reduction over 12 months

The CE analyses showed that UC+SVAI was dominant compared to UC (table 4). Table 4 and supplementary figure 5 show that most incremental CE pairs for sickness absence were located on the southeast quadrant of the CE plane, indicating that UC+SVAI was on average less costly and more effective.

Both CE acceptability curves show that the probability of UC+MI and UC+SVAI being cost-effective compared to UC exceeds 90% across all willingness-to-pay thresholds (supplementary figure S4 and S5 a and b).

### Cost-benefit analysis

Results of the cost-benefit analyses were in favor of MI+UC (table 5). The total benefit was €5278 (95% CI -562–10 999) in the MI+UC compared to the UC group. The mean net benefit was €5225 (95% CI -592–10 985) per worker. BCR (ie, amount of money returned per euro invested) was €99 (95% CI -10–214). The estimated maximal probability of return was 96.3%, indicating 96.3% probability for NAV to expect a positive ROI from the intervention.

**Table 5:** Return-on-Investment analysis results (main analysis). [CI=confidence interval; UC=usual case management; MI=motivational interviewing; SVAI=stratified vocational advice intervention].

Sample size		N	Costs € (95% CI)	Benefits total € (95% CI)	Net benefit € (95% CI)	Benefit cost ratio € (95% CI)
Comparison 1: UC+MI vs. UC			53 (48–59)	5278 (-562–10 999)	5225 (-592–10 985)	99 (-10–214)
	MI+UC	169				
	UC	171				
Comparison 2: UC+SVAI vs. UC			78 (72–85)	7293 (1603–12 884)	7214 (1548–12 851)	93 (21–166)
	SVAI+UC	169				
	UC	171				

Results of the cost-benefit analyses were also in favor of SVAI+UC (table 5). The total benefit was €7293 (95% CI 1603–12 884) in the SVAI+UC compared to the UC group, with a mean net benefit of €7214 (95% CI 1548–12 851) per worker. The BCR was €93 (95% CI 21–166). The estimated maximal probability of return was 99.5%, indicating 99.5% probability for NAV to expect a positive ROI from the intervention.

### Sensitivity analyses

In analyses of sickness absence days, imputed, adjusted analyses showed for UC+MI versus UC -13.4 sickness absence days (95% CI -25.0–1.1) and for UC+SVAI versus UC -13.9 (95% CI -8.0–0.6) sickness absence days. Replicating the CE analyses using complete case analysis, and the unimputed analysis yielded results consistent with the primary findings (data not shown).

## Discussion

Twelve-month results for individuals on sickness absence due to musculoskeletal disorders showed that adding MI or SVAI to UC significantly reduced the number of sickness absence days, sickness absence benefits, and the interventions were cost-effective from a societal perspective. The intervention groups had lower odds for receiving wage replacement benefits during the 12-month follow-up compared to UC. The probability of UC+MI and UC+SVAI being cost-effective compared to UC was >90% across all willingness-to-pay thresholds. The financial return estimates were likely to be positive for MI and highly positive for SVAI.

To identify possible mechanisms of the interventions, we investigated the mediating effects of “RTW expectations” and “workability” at the 6-month follow-up (19). The mediation analyses showed that changing an individual’s RTW expectations reduced sickness absence, indicating that MI and SVAI might be particularly effective for those with low RTW expectations. Furthermore, our treatment effect modifier analysis showed that analgesic medication use (SVAI), age and self-perceived health (MI) may potentially modify the effect of these two interventions (20). Norway has a generous welfare system that provides full wage replacement for up to one year following the onset of sick leave. At the same time, the system includes mechanisms intended to support work participation, such as the encouragement for GP to prescribe part-time sick leave when appropriate. This enables individuals to maintain a connection to the workplace while recovering. Nonetheless, the generous benefits may unintentionally discourage early or accelerated RTW for some individuals—particularly in cases where sick leave is driven by work-related factors such as workplace conflicts, poor working conditions, or high job demands.

Another point is that even though MI and SVAI were low-intensity interventions, both, and particularly SVAI, might have generated additional treatment, such as seeking more healthcare or other help. However, even though contacting other stakeholders was included in the SVAI protocol, the SVAI physiotherapist had limited liaison with other stakeholders. Furthermore, the MI and SVAI groups had lower healthcare costs compared to the UC group, indicating that they received less healthcare.

### Comparison with previous research

Among participants with musculoskeletal disorders on sick leave for ≥7 weeks, both the 6-month (14) and 12-month results showed effectiveness of adding the interventions to UC. The interventions might be more effective for subgroups of the participants. Our results partly differ with those of Aasdahl et al (35) whose recent large RCT in Norway showed no 12-month differences in sickness absence days or time-to-sustained RTW between UC+MI compared to UC or an active control group. Notably, their RCT included individuals on sick leave with any diagnosis, 42% had musculoskeletal disorders, in contrast to our study which included participants with musculoskeletal disorders only. Furthermore, only half of the participants in Aasdahl and colleagues’ MI group received the intervention, compared to 70% in our trial. In addition, their interventions were delivered after 4 months of sickness absence, potentially influencing the effect estimates. A pilot RCT from Belgium (36) tested if a 15-minutes MI consultation, delivered by a MI certified clinical psychologist after 3–6 months of work disability, could improve RTW. The results showed that participants receiving MI had faster RTW and lower risk of relapse compared to consult as usual. Around half of the participants had musculoskeletal disorders, chronic pain or fatigue. Twelve-month results from a cluster RCT conducted in a vocational rehabilitation facility in Canada also showed that adding MI to UC improved RTW and reduced wage replacement benefits among claimants on sickness absence with musculoskeletal conditions (37, 38). This study included people undergoing rehabilitation for musculoskeletal conditions who were predominantly employed and received the MI intervention during the rehabilitation. Although there are still relatively few studies, MI seems to be a promising intervention to assist people with musculoskeletal disorders returning to work.

The SVAI intervention showed promising effectiveness on RTW outcomes and costs. The SVAI is an adapted version of the intervention in the UK Study of Work and Pain (SWAP) trial (13). We modified and tested the intervention and found that adding 1–4 phone calls from physiotherapists to UC reduced sickness absence by almost 18 days over 12 months, corresponding to 3.5 work weeks. In contrast, the SWAP trial, which tested a vocational advice service for patients with musculoskeletal disorders in primary care, found no difference in the overall number of sickness absence days at 12-month follow-up. However, the intervention group reported significantly fewer GP-certified days off work: 16.4 (SD 34.2) compared to 22.9 (SD 50.5) days in the control group. The SWAP trial differed from our study because in the latter everyone had ≥50% sick leave whereas the SWAP trial included those absent from or struggling to remain in work. Few studies have examined cost-benefit analyses of such interventions beyond the SWAP trial (13), which found that adding vocational advice in UK primary care was cost-saving for society compared to usual care alone.

Addressing both health and work-related obstacles and collaborating with the workplace could be an even more efficient way to facilitate RTW for people on sickness absence with musculoskeletal disorders. Our results showed no difference in self-reported musculoskeletal health between the intervention groups, suggesting the need for additional interventions to improve health.

### Strength and limitations

This study is among the first to report CE analyses and results in this field adding important knowledge to the research field and stakeholders working with sick leave follow-up. We had high-quality registry data on 99% of the study participants covering sickness absence outcomes, ensuring little missing values. The trial included an internal pilot phase to assess recruitment and feasibility of the interventions, and fidelity evaluations of the delivery of the MI and SVAI. However, only 25% of the eligible participants consented to participate in the trial, possibly reducing the generalizability of the results. We do not have information about the reasons for non-participation. Furthermore, many participants never got the intervention (figure 1), for some explained by early RTW. There was missing data for some baseline variables. Hence, we conducted analyses with multiple imputations. Wide CI indicates low precision of the effect estimates. Finally, the RCT had no attention control group. Therefore, we do not know if the interventions worked only as a result of the extra professional attention.

Our findings indicate that the low-intensity interventions MI and SVAI are cost-effective. These results have important implications for policy: MI should be considered for implementation as a tool for case workers at NAV offices across Norway, and healthcare providers involved in sick leave follow-up should receive training in SVAI to enhance support and outcomes for individuals on sick leave. Furthermore, additional research into the mechanisms of action underlying these interventions is needed to optimize their effectiveness and better target specific subgroups.

### Concluding remarks

This trial demonstrated that adding either MI or SVAI to UC significantly improved RTW outcomes at 12 months and was cost-effective compared to UC alone for individuals on sick leave due to musculoskeletal disorders. These low-intensity interventions could reduce the burden of long-term sickness absence for individuals with musculoskeletal disorders.

## Supplementary material

Supplementary material
