# Supplementary material for: Effectiveness of two vocational interventions on sickness absence and costs for people with musculoskeletal disorders: 12 months results from the MI-NAV multi-arm randomized trial
Source: Scand J Work Environ Health. 2025 Oct 30;51(6):505–15. doi: 10.5271/sjweh.4248 (PMC12591726; doi:10.5271/sjweh.4248)
Supplement: Supplementary material [file SJWEH-51-505-S001.pdf]

Effectiveness of two vocational interventions on sickness absence and costs for people with musculoskeletal disorders: 12 months results from the MI-NAV multi-arm randomized trial[1]

by Britt Elin Øiestad, PhD,[2] *Esther Maas, PhD, Fiona Aanesen, PhD, Alexander Tingulstad, PhD, Tarjei Rysstad, PhD, Maurits van Tulder, PhD, Anne Therese Tveter, PhD, Milada Hagen, PhD, Rigmor C Berg, PhD, Nadine E Foster, PhD, Gwenllian Wynne-Jones, PhD, Gail Sowden, PhD, Gunnhild Bagøien, PhD, Roger Hagen, PhD, Kjersti Storheim, PhD, Margreth Grotle, PhD*

1. Supplementary material
2. Correspondence to: Britt Elin Øiestad, Department of Rehabilitation science and health technology, Oslo Metropolitan University, P-O. Box 4, St. Olavs Plass, 0130 Oslo, Norway. [E-mail: brielo@oslomet.no].

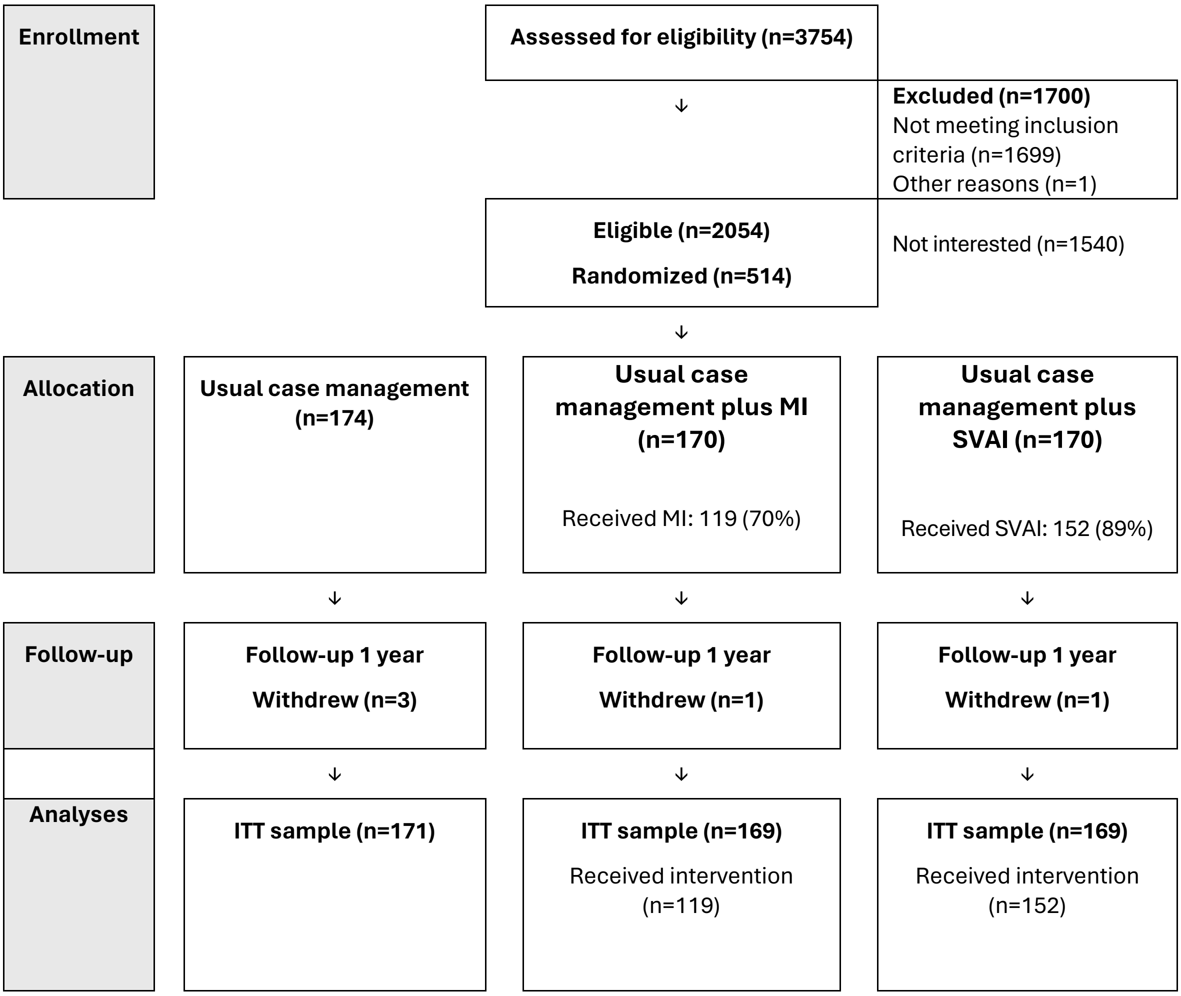

Figure S1. Flow diagram of study participants in the randomized controlled trial.

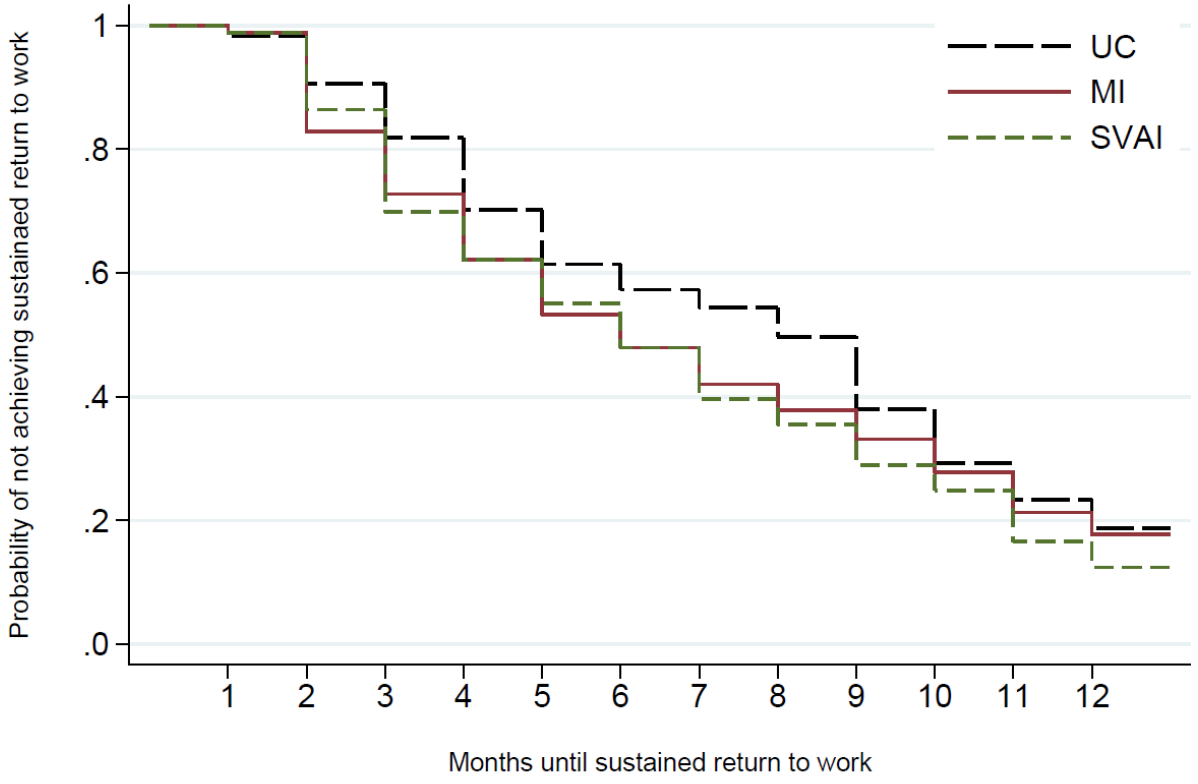

Figure S2. Probability of participants with sustained return to work in the three groups over 12 months. UC: usual case management, MI: motivational interviewing, SVAI: stratified vocational advice intervention.

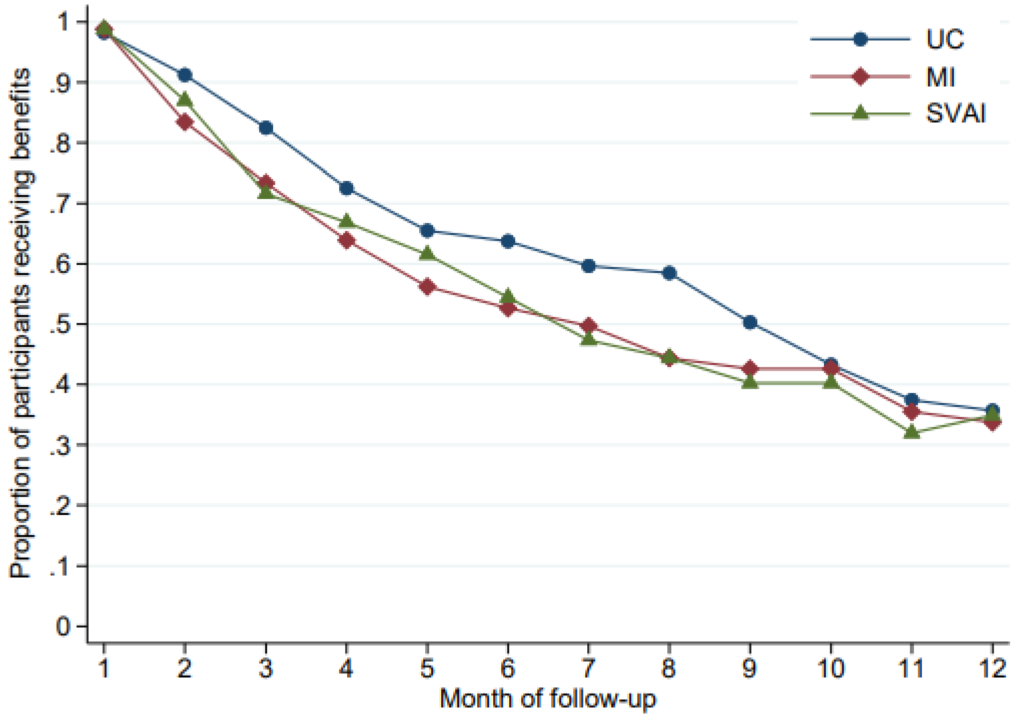

Figure S3. Proportions of participants in each group receiving wage replacement benefits each month during the 12-month follow-up period. UC: usual case management, MI: motivational interviewing, SVAI: stratified vocational advice intervention.

A

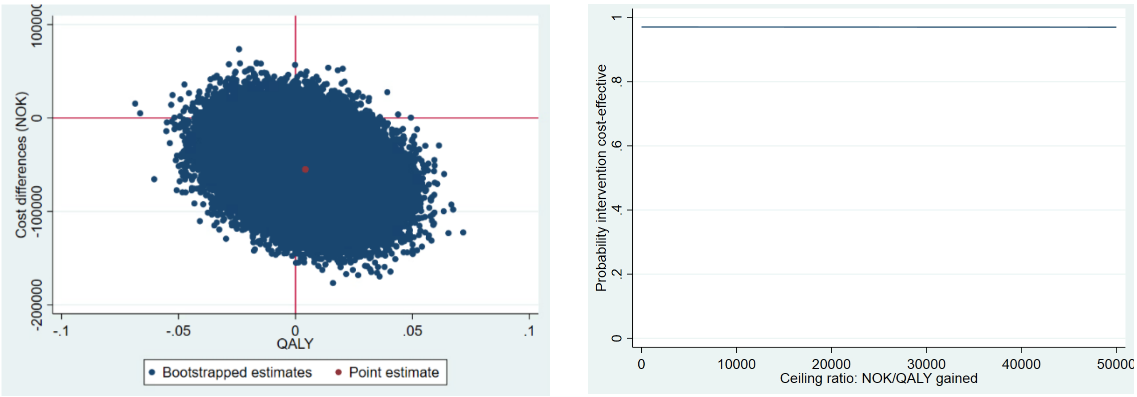

B

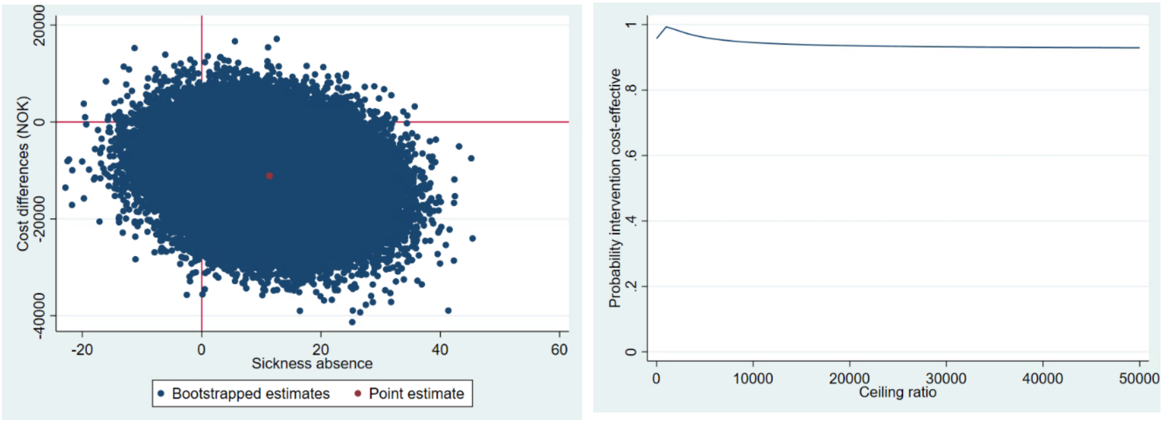

Figure S4. Cost-Utility plane & Cost-Utility acceptability curve for different ceiling ratios (NOK) for quality-adjusted life-years indicating the probability of cost-effectiveness of Motivational Interviewing versus control for workers on (a) QALYs or (b) sickness absence due to a musculoskeletal disorder.

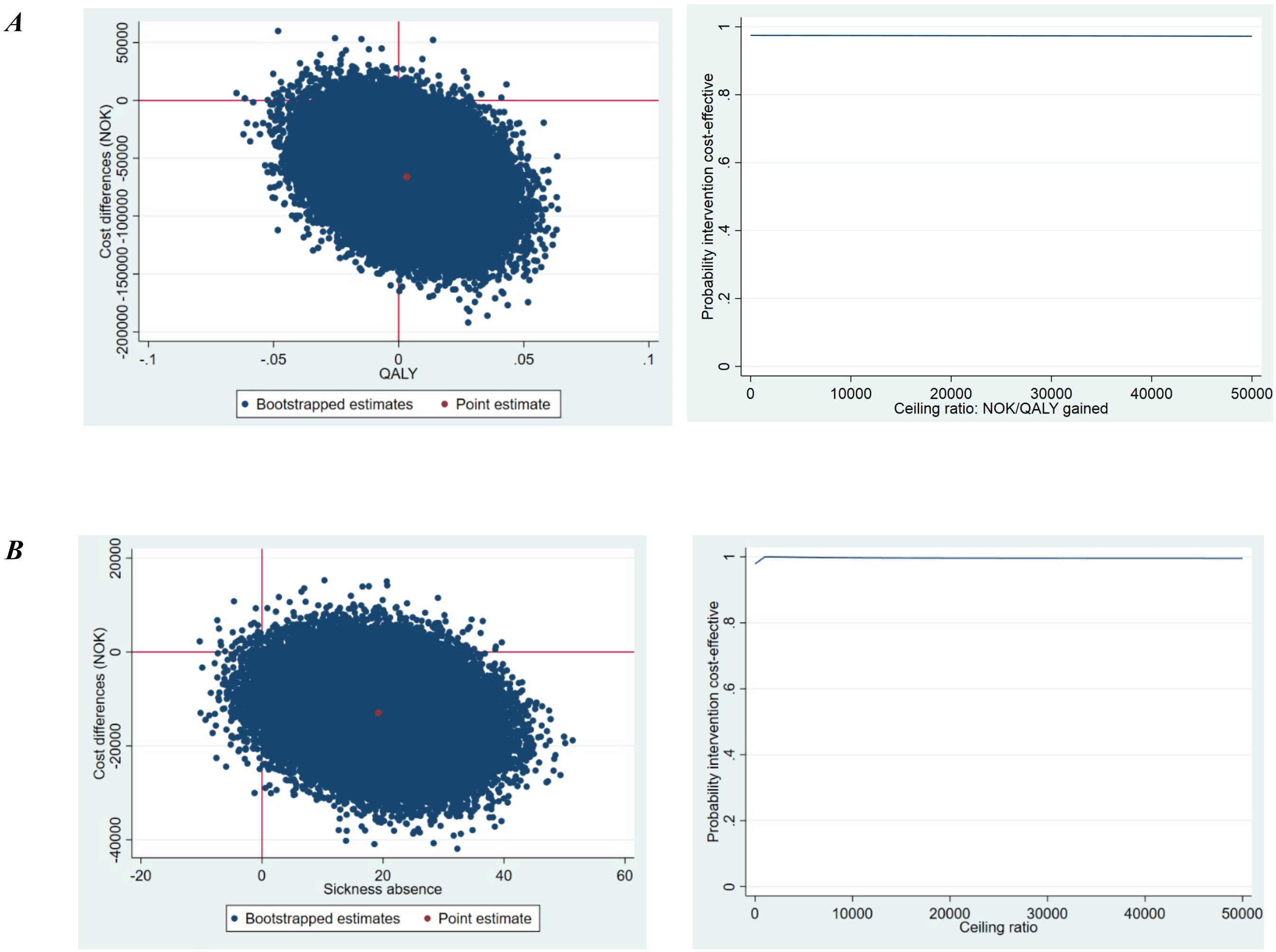

Figure S5. Cost-Utility plane & Cost-Utility acceptability curve for different ceiling ratios (Norwegian Kroner) for quality-adjusted life-years indicating the probability of cost-effectiveness of Stratified Vocational Advice Interventions (SVAI) versus control for workers on (a) QALYs or (b) sickness absence due to a musculoskeletal disorder.
